# Keeping It Together: Structures, Functions, and Applications of Viral Decoration Proteins

**DOI:** 10.3390/v12101163

**Published:** 2020-10-14

**Authors:** Corynne L. Dedeo, Carolyn M. Teschke, Andrei T. Alexandrescu

**Affiliations:** Department of Molecular and Cell Biology, University of Connecticut, 91 North Eagleville Road, Unit-3125. Storrs, CT 06029-3125, USA; Corynne.dedeo@uconn.edu

**Keywords:** structure-function relationships, virus surfaces, bacteriophage, host-pathogen interactions, quasi-symmetry, innate immunity, biomimetics, nanomedicine

## Abstract

Decoration proteins are viral accessory gene products that adorn the surfaces of some phages and viral capsids, particularly tailed dsDNA phages. These proteins often play a “cementing” role, reinforcing capsids against accumulating internal pressure due to genome packaging, or environmental insults such as extremes of temperature or pH. Many decoration proteins serve alternative functions, including target cell recognition, participation in viral assembly, capsid size determination, or modulation of host gene expression. Examples that currently have structures characterized to high-resolution fall into five main folding motifs: β-tulip, β-tadpole, OB-fold, Ig-like, and a rare knotted α-helical fold. Most of these folding motifs have structure homologs in virus and target cell proteins, suggesting horizontal gene transfer was important in their evolution. Oligomerization states of decoration proteins range from monomers to trimers, with the latter most typical. Decoration proteins bind to a variety of loci on capsids that include icosahedral 2-, 3-, and 5-fold symmetry axes, as well as pseudo-symmetry sites. These binding sites often correspond to “weak points” on the capsid lattice. Because of their unique abilities to bind virus surfaces noncovalently, decoration proteins are increasingly exploited for technology, with uses including phage display, viral functionalization, vaccination, and improved nanoparticle design for imaging and drug delivery. These applications will undoubtedly benefit from further advances in our understanding of these versatile augmenters of viral functions.

## 1. Introduction

Viruses and bacteriophages (phages) have their genetic material enveloped by membranes or encapsulated in proteinaceous shells called capsids. The stabilities of the frameworks that harbor the nuclei acid genomes are crucial for the successful assembly and infectivity of phages and viruses [1]. Tailed phages (*Caudoviralaes*) together with related viruses including herpesviruses, adenoviruses, and some archaeal and giant viruses, initially assemble immature meta-stable structures called procapsids. These undergo irreversible conformational changes upon DNA packaging to become mature capsids. An increase in volume often accompanies the process, and requires stabilization of the capsid against the buildup of internal pressure that results from genome packaging [2,3,4,5]. Stabilization of some capsids is achieved through amino acid modifications that covalently cross-link coat protein subunits, others make use of interactions between genetically inserted auxiliary domains within coat proteins (I-domains), while some have their capsids stabilized by accessory proteins [2,4,6,7,8,9].

Accessory proteins are broadly defined as virally encoded proteins that do not have a role in virus replication but rather modify the properties of the virion [10]. These proteins usually bind capsids in the final stages or after completion of viral assembly, and play a variety of roles in the infection cycle, including stabilizing the expanded capsid and modulating host interactions [11]. Among the accessory proteins are decoration [11] and cementing proteins [12]. The nomenclature has become largely interchangeable; although in the original definitions decoration proteins were designated as binding to the surfaces of mature virions [11,13], whereas cementing proteins had the additional feature of stabilizing the virion upon binding [12]. The distinction is tenuous because it presupposes measuring the effects of the accessory protein on virion stability, which is often more difficult than identifying a viral surface protein. Indeed, many of the decoration proteins for which data are available such as Dec (L) [13], gpD (λ) [14], gp56 (TW1) [15], pb10 (T5) [16], gp87 (P74-26) [17], gp88 (P23-45) [18] and Soc (T4) [19] stabilize viruses, making them operationally indistinguishable from cementing proteins. We therefore use the term “decoration proteins” for this review. For brevity, we use a naming scheme in which the decoration protein is listed first, followed by the virus that encodes it in parentheses. For example, gpD (λ) refers to the decoration protein gpD from phage λ.

To date, decoration proteins are only known to occur in viruses with DNA genomes [17]. Within the DNA viruses, they are common and span many genera including tailed phages (*Caudovirales*) [13,15,20], herpesviruses [21,22,23], adenoviruses [12,24,25,26], as well as archaeal viruses [17,27,28], and giant viruses [29,30,31]. They appear to be particularly common in tailed phages, which “inject” their DNA genomes, and are thus under no selective pressure to break their capsids during infection [28]. The ubiquity of decoration proteins might be underestimated. For many viruses, surface protrusions may be initially imaged only at low-resolution, so that the presence of decoration proteins could be missed until the virus is better characterized. The capsid structures of three actinobacteriophages, discovered through a collaboration with the SEA-PHAGES (Science Education Alliance-Phage Hunters Advancing Genomics and Evolutionary Science) program, were recently resolved to approximately 6 Å by cryo-EM. Of these phages, two had novel decoration proteins with no known sequence homologs [32]. Similarly, 7 of 16 newly discovered *Shigella*-infecting phages had a novel decoration protein [33]. Finally, also recently described are structures of the jumbo phages G at 6 Å [34] and ΦRSL2 (16 Å) [35], and the semi-jumbo phage ΦRP13 (9.5 Å) [35], each of which have decoration proteins, though the resolution of these reconstructions precludes knowing if they are unique decoration proteins. Although this abundance of decoration proteins may be due to their prevalence in nature, it could also reflect a partial bias in collection and isolation methods, as phages with decoration proteins are often more resistant to harsh environmental conditions.

Since a comprehensive review of decoration proteins is a daunting task, we focus here on the subset that have high-resolution structures and well-established functions. Much of our knowledge about decoration proteins owes a large debt to the groundbreaking work of Michael Rossmann [15,19,36,37,38,39,40], whose contributions and accomplishments are celebrated in this special issue of Viruses. In this review, we discuss the versatile functions of decoration proteins, classify and analyze their structures and virus binding sites, and explore the opportunities these proteins present for nanotechnology applications.

## 2. Functions of Decoration Proteins

Although most decoration proteins contribute to capsid stability and maturation, some have additional roles such as mediating viral targeting of host cells. The functional properties of decoration proteins are reviewed in this subsection with an overview provided in Table 1.

### 2.1. Stabilization of Capsids by Decoration Proteins

A common function of decoration proteins is to increase virus stability. The phage HK97 (Hong Kong 97) coat protein fold is prevalent across dsDNA viruses [41]. The commonality of this structural motif is thought to be due to its ability to form a variety of icosahedral and prolate capsids, spanning a range of sizes [31,42]. The prototypical example, from phage HK97, features covalent cross-links between capsomers across icosahedral 3-fold symmetry axes, as well as between subunits within capsomers. [2,42,43]. This “chainmail” of covalent links reinforces the capsid against internal pressure. The dsDNA genome of HK97 is packaged to liquid crystalline density, with the ensuant pressure comparable to that of a pressurized champagne bottle [14,44,45].

To withstand internal pressure from genome packaging or external environmental insults, viruses lacking the ability to form cross-links have evolved several strategies to strengthen their capsids, including encoding stabilizing decoration proteins [6,14,46]. In some cases, such as phage λ, decoration proteins are required for maturation but the majority of decoration proteins only function to stabilize capsids, suggesting they may confer a selective advantage only under conditions of viral stress [14,47].

#### 2.1.1. Decoration Proteins are Required for the Assembly of Some Viruses

An example of a decoration protein necessary for phage assembly is gpD (λ). gpD (λ) binds to expanded capsids of λ phage during the last stages of DNA packaging, to stabilize them while the last 10–20% of the genome needed for maturation is incorporated [49,74,75]. The cementing function of gpD (λ) in λ phage has been suggested to substitute for the covalent cross-links that form the “chainmail” structure of HK97 phage [14,49]. Atomic force microscopy (AFM) experiments indicate that gpD (λ) additionally reinforces the λ virus against mechanical pressure and collisions [48]. gpD (λ) is related to decoration proteins from other λ-like phages, including SHP (21), which shares 49% sequence identity. Interestingly, SHP (21) can bind to the λ capsid as a chimeric oligomer with gpD (λ), producing particles with varying stabilities [76]. Decoration proteins gp56 (TW1), gp87 (P74-26) along with its close relative gp88 (P23-45), and gp8.5 (φ29), as well as the Herpes Simplex Virus-1 (HSV-1) VP23/VP19C and Human Cytomegalovirus (HCMV) Tri1/Tri2 triplex proteins [21,22,23], share both the β-tulip fold and capsid-stabilizing function of gpD (λ). If these related proteins are also required for the assembly of their respective phages and viruses is currently unknown [15,17,40,77].

Herpesviruses share similarities with dsDNA phages that extend to structures and assembly mechanisms [78]. The coat protein of herpesviruses is based on the HK97-fold, and triplex proteins have the same β-tulip fold found in decoration proteins such as gpD (λ), gp87 (P74-26) and gp88 (P23-45). Similar to gpD (λ), the triplex proteins are necessary for capsid assembly but because of the larger genome, additional proteins are also used to reinforce herpesvirus capsids [79,80,81].

#### 2.1.2. Some Decoration Proteins Provide Stability but Are Not Required for Infectivity

Although some decoration proteins are necessary for virus maturation, others function primarily to boost stability. In contrast to gpD (λ), Dec (L) is not required for phage infectivity. In addition to its natural substrate phage L, Dec (L) can also noncovalently bind and stabilize expanded heads or mature capsids of phage P22 in vitro and in vivo [13,47]. This occurs because the coat proteins of phages L and P22 are highly homologous, differing in only 4 out of 430 positions (99.6% identical) [13,57]. P22 is often substituted as a model for phage L, owing to its extremely well-characterized genetics and biochemistry [47]. Dec (L) stabilizes phage P22 in the presence of EDTA, a chelator that binds Mg^2+^. In the absence of Mg^2+^, the dsDNA genome of P22 undergoes decondensation that causes the phages to burst because of the increase in internal pressure. Without Dec (L), 90% of P22 phages are destroyed in the presence of EDTA. By contrast, EDTA has no effect on P22 phages when these are bound by Dec (L) [13]. Despite not being essential for phage viability, Dec (L) clearly plays a role in stabilizing the phage L capsid against internal pressure, and may facilitate the survival of the phage in harsh environments.

Similar to Dec (L), gp17 (N4) does not affect infectivity but offers stability under harsh conditions, including exposure to DNase I and ETDA [19]. Soc (T4) found in T4 and T4-like phages, together with pb10 (T5) [16] and gp10 (ε15) [73] are expendable for maturation and infectivity but stabilize their respective capsids in their matured states. Pb10 (T5) prevents DNA leakage under low ionic strength conditions, while Soc(T4) stabilizes the capsid against temperature and pH extremes [16,39,58].

Taken together, raised stability appears to be a key function for decoration proteins. In cases where decoration proteins are expendable for phage viability, their stabilizing effects are likely beneficial when the virus is subjected to stress [16,47,58,59].

### 2.2. Multifunctional Decoration Proteins

Viruses are under selective pressure to economize their genomes. Addition of a stabilizing protein could require a larger capsid to accommodate the concomitant increase in genetic material encoding the new protein. Consequently, it is perhaps not surprising that decoration proteins are often multifunctional, augmenting their roles in capsid stabilization with additional moonlighting functions that include acting as viral tape-measures for capsid size or serving as mRNA transcription anti-terminators.

#### 2.2.1. Decoration Proteins that Act as ‘Tape-Measures’ to Determine Virus Size

Phage PRD1 is evolutionarily related to complex viruses such as adenovirus and the giant virus PCBV-1 [37,82,83]. All these feature decoration proteins that function as both cementing stabilizers and “tape-measure” regulators of capsid size.

The decoration protein P30 (PRD1) is necessary for phage maturation and additionally forms a cage-like structure that anchors the capsid to the internal membrane encapsulating the genome [70,71]. P30 (PRD1) modulates the capsid transformations required for assembly, and has been compared to both scaffolding and tape-measure proteins, as it governs both nucleation and capsid size determination [70].

Adenovirus features four cementing proteins (IIIa, VI, VIII, and IX) that are proteolytically cleaved during the virus maturation process. The primary function of all four proteins is to buttress capsid stability by connecting capsomers both internally and externally. Some of the four proteins play additional roles in the infection cycle [12,24]. IIIa (adenovirus) has been suggested to act as a tape-measure protein, similar to P30 (PRD1) [25]. The mature form of VI (adenovirus) has an N-terminal amphipathic β-helix that allows the virus to escape from endosomes during the infection process [24].

The giant *Paramecium bursaria* chlorella virus, PCBV-1, has a staggering 13 minor capsid proteins (P2–P14) that provide structural stability. The minor capsid proteins cement capsomers in triangular and pentameric arrangements, known as symmetrons [37]. In addition, the minor capsid proteins P12, P13, and P14 anchor the external capsid to the internal membrane encapsulating the nucleocapsid [37,72]. The PCBV-1 minor protein P2, in addition to providing structural support, acts as a tape-measure that controls the size of the giant PCBV-1 virus [37].

#### 2.2.2. The Psu Decoration Protein Moonlights as a Transcription Antiterminator

Polarity suppression protein Psu (P4) is a non-essential but multifunctional decoration protein in phage P4. On the capsid surface, Psu (P4) forms V-shaped dimers that cover structural gaps at the centers of hexons in the icosahedral lattice [64]. The dimerization interface of Psu (P4) forms a knotted and highly hydrophobic structure that likely imparts the decoration protein-capsid complex significant tensile strength [64]. In the infected cell, Psu (P4) additionally functions as a transcription antiterminator by inhibiting the host transcription termination factor, Rho [64,65].

### 2.3. Participation of Decoration Proteins in Host Attachment

Several decoration proteins, including Hoc (T4), pb10 (T5), gp12 (SPP1), and gp8.5 (φ29), contribute little to capsid structural integrity but rather enhance binding to both host and non-host cell surfaces through interactions with carbohydrates [40,60,61,63,66,67]. Mediation of these interactions by decoration proteins can serve two purposes. First, the decoration proteins can concentrate phages to the bacterial target cells, aiding their infectivity [84], or conversely disperse phages through electrostatic surface repulsion preventing their aggregation [38]. Second, binding of decoration proteins to glycans emanating from the mucus layers of metazoan cells may facilitate a symbiotic form of non-host innate immunity [85,86]. In animal cells, the mucus layer, which is rich in complex glycoproteins and antimicrobial compounds, is part of the innate immune system that forms the first line of defense against infection [85,87]. Phages can aggregate in the mucus layer via decoration protein mediated glycan binding, where they can protect animal cells by lysing invading bacteria [38,84,85,86,88].

#### 2.3.1. Decoration Proteins with Ig-Like Domains Can Participate in Host Adhesion

A bioinformatics survey showed that 25% of tailed dsDNA phages encoded proteins with predicted immunoglobulin-like (Ig-like) β-sandwich folding motif [89]. These domains were found in five functional classes: tail fibers, baseplate wedge initiators, major tail components, major head components (such as coat protein insertion domains), and decoration proteins [89]. Highly immunogenic outer capsid proteins, such as Hoc (T4), pb10 (T5), and gp17 (N4) share an Ig-like fold, and are thought not to contribute significantly to capsid stability but instead to mediate host cell adhesion [19,38,61,63]. T4 particles missing Hoc (T4) tend to aggregate at low cation concentrations, indicating that the decoration protein could also be important for dispersal of viral particles when the host cell density is low [38].

#### 2.3.2. Head Fibers May Coordinate Cell Attachment

Like the Hoc (T4) decoration protein, head fibers do not affect capsid stability or infectivity of the φ29 phage [40]. Phage φ29 particles decorated with head fibers, however, interact in an ordered fashion with host cell bacterial walls compared to fiber-less phages. This suggests head fibers may aid infectivity under conditions of low viral presence [40]. Although most decoration proteins are rich in β-sheet structure, the φ29 head fiber is comprised of an elongated α-helical coil-coiled, similar to that found in the T4 fibritin and P22 tail needle structures [40]. As these proteins are important for attachment to target cells, it is possible that the φ29 head fiber is evolutionarily related to them. Likewise, some proteins predicted to be collagen-like, such as those in the elongated trimeric spike gp12 (SPP1), may be important for cell surface recognition [66,67,68].

## 3. Decoration Protein Structures

Decoration proteins show considerable structural versatility both in terms of the folds they adopt as well as their capsid-binding mechanisms. In this subsection we review the main structural motifs of decoration proteins, their capsid-binding modes, and possible evolutionary relationships suggested by structural homology.

### 3.1. Capsid-Binding Modes and Oligomerization States

**Figure 1:**
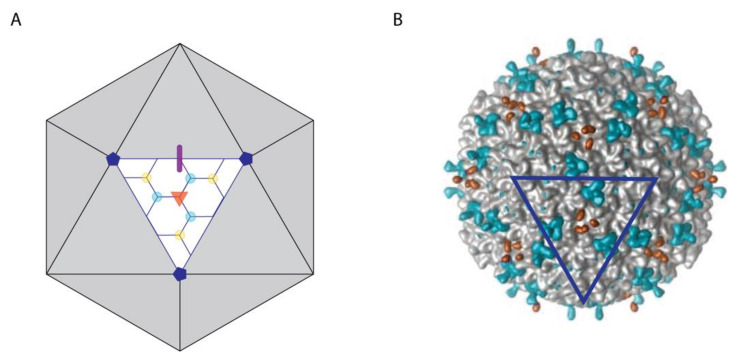
Symmetry of capsid-binding sites for decoration proteins. (**A**) Model of a *T* = 7 icosahedral capsid with symmetry sites highlighted on one facet. The 2-, 3-, and 5-fold symmetry axes are shown by a thick purple line, an orange triangle, and dark blue pentagons, respectively. Additionally, two types of quasi-3-fold axes are indicated. The first connects only hexons and is shown by cyan dots, the second connects two hexons and a penton and is shown using yellow dots. (**B**) Surface map of the phage L capsid bound by the decoration protein Dec (L) at 3-fold (orange) and type I quasi-3-fold (cyan) sites, corresponding to those illustrated in panel A. The dark blue triangle outlines a facet in the same orientation as in panel A. The figure is adapted from Tang et al. [47].

Decoration proteins bind on the surfaces of viruses as exemplified by the cryo-EM image of Dec (L) in Figure 1. As such, decoration proteins can have significant effects on the ruggedness and patterning of the viral surface [9], which in turn can affect virus recognition, including interactions with target cells or host defenses.

#### 3.1.1. Decoration Proteins Bind to a Variety of Symmetry and/or Pseudo-Symmetry Axes

The icosahedral frameworks of spherical and prolate capsids have a basis set of 2-, 3-, and 5-fold symmetry axes, as summarized in the schematic of Figure 1A. In addition to these true symmetry axes, there exist quasi-three-fold sites [57]. The first type occurs between hexamers on icosahedral facets as indicated by the cyan dots in Figure 1A. A second type of quasi-three-fold site lies between pentons and hexons surrounding each vertex, as indicated by yellow dots Figure 1B. The differences between three-fold (orange) and quasi-three-fold (cyan, yellow) sites is illustrated with the cryo-EM [47] surface map of Dec (L) bound to phage L in Figure 1B. A summary of capsid-binding-site symmetries for different types of decoration proteins is given in Table 1.

Most decoration proteins bind to three-fold and quasi-three-fold symmetry axes [14,15,28]. These are the sites reinforced by covalent cross-links in the HK97 capsid [41], and thought to correspond to weak points in the icosahedral lattice [28,90]. Consistently the three-folds sites, which occur between the icosahedral hexamers, have been shown to be mechanical weak points susceptible to bursting at increased internal DNA pressure in modeling studies [91]. There are, however, decoration proteins with alternative preferential binding sites. Thus, P30 (PRD1), for example, is a proline-rich, mostly disordered, extended protein that when capsid-bound dimerizes through an N-terminal hook at the icosahedral 2-fold axis of symmetry [70]. The network of dimers forms a chainmail-like structure surrounding the capsid ~12 Å above the viral membrane [70].

Several decoration proteins bind at both true and quasi-symmetry sites, or prefer one type of site over others. For example, Dec (L) binds type I quasi-three-fold sites between hexons 1000 times more strongly than true three-folds [92]. Structural data from cryo-EM suggests that Dec (L) discriminates binding-site topologies by forming a larger number of contacts with the higher avidity quasi-three-fold site [57]. By contrast, trimers of YSD1_16 (YSD1) create a non-covalent chainmail-like structure that includes binding sites at both three-fold and quasi-three-fold symmetry axes [54]. Both Soc (T4) and Soc (RB69), from phage RB59 a close relative of T4, bind their respective capsid as trimers. The tadpole-like heads of the Soc decoration proteins point to quasi-two-fold axes relating adjacent hexamers, and their tails are located near quasi-three-fold axes [39]. Trimers of gp8.5 (φ29) bind the mature capsid at quasi-3-fold axes of symmetry where they interact with the Ig-like coat protein insertion domain, BIG2 [77]. The elongated decoration protein gp17 (N4) comprised of three Ig-like domains [19], binds as a monomer to both types of quasi-three-fold axes (cyan and yellow in Fig 1B). By contrast, the decoration protein Hoc (T4), which also has an elongated shape consisting of three Ig-like domains and an N-terminal capsid-binding domain, binds as a monomer preferentially to the quasi-6-fold axis at the center of hexons [61]. Adding to the wide diversity of decoration protein structures are those that are α-helical, including Psu (P4) [64] and gp12 (SPP1) [66,67]. Both proteins feature coiled-coil structures that bind to the center of hexons, corresponding to a quasi-6-fold symmetry axis.

Rather than showing a unifying capsid-binding theme, the interactions between decoration proteins and capsids seem to be structurally opportunistic, employing a wide variety of binding modes that depend on the distinct structures of the decoration proteins and of the coat proteins that make up the cognate capsids.

#### 3.1.2. Oligomerization of Some Decoration Proteins May Require Capsid Binding

The oligomerization states of capsid-bound decoration proteins are summarized in Table 1. Known examples include monomers, dimers, and trimers, with the latter being the most common. Most recent structural models of decoration proteins have come from cryo-EM studies, where only the capsid-bound state is determined. To have information on both the capsid-bound and -unbound structures is much rarer; however, these data exist for gpD (λ), SHP (21), Dec (L), and Soc (RB69). In three of four cases the decoration protein is a trimer when capsid-bound but can exist as a monomer in solution. Soc is a monomer in solution by analytical ultracentrifugation [62] and only becomes a trimer when capsid-bound [39]. Similarly, gpD (λ) is a monomer and only trimerizes on capsids, or in crystals [50,51]. Dec (L) forms a monomer in solution upon acidification that consists of a folded N-terminal domain and a disordered C-terminal tail [56]. The C-terminal tail is thought to fold into a three-stranded α-helix structure in the capsid-bound state [57], since if the tail is deleted Dec(L) can no longer bind capsids (ATA and CMT, unpublished observation). These observations raise the question of whether some decoration proteins only oligomerize in their capsid-bound states. By contrast SHP(21), which is homologous to gpD (λ), is a trimer in both solution and capsid-bound states [52].

### 3.2. Current Decoration Protein Structures Fall into Five Main Folding Motifs

Protein structure is arranged hierarchically. Segments of hydrogen-bonded secondary structure such as α-helices and β-strands coalesce into higher-order “super-secondary structure”—a concept originated by Michael Rossmann [93]. These super-secondary structure modules can govern tertiary folding topology, which often provides clues about the functions and evolutionary relationships of proteins. For example, the Rossmann-fold—one of the most ubiquitous protein folds in nature (named after Michael Rossmann)—is an α/β structure that is typically found in proteins and enzymes with nucleotide-binding functions [94,95].

**Figure 2:**
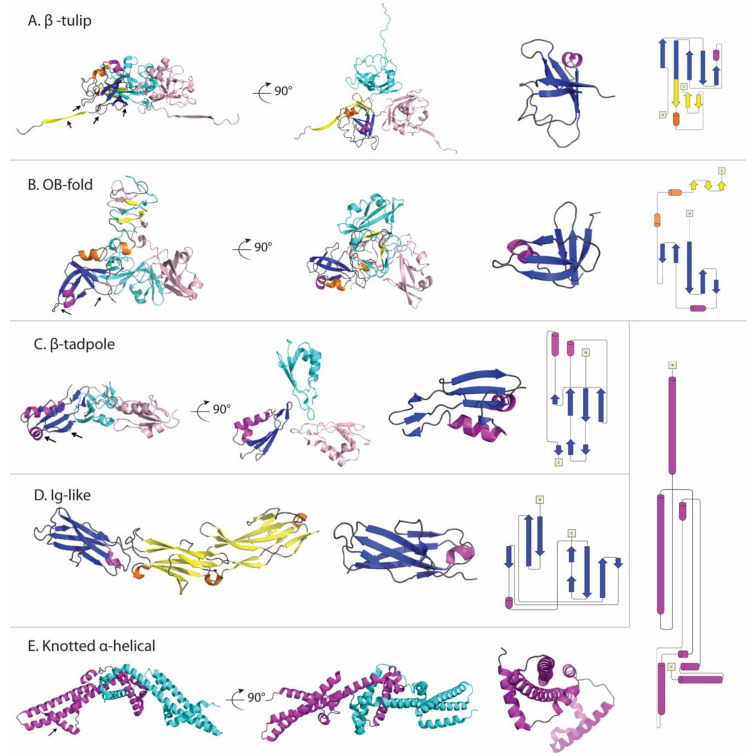
Comparison of decoration protein structures. (**A**) β-Tulip fold illustrated by gp87 (P74-26), PDB 6O3H. (**B**) OB-fold in Dec (L), PDB 6E3C. (**C**) β−Tadpole fold in Soc (T4), PDB 3IG9. (**D**) Ig-like fold exemplified by Hoc (T4), PDB 3SHS. (**E**) Knotted α-helix fold shown by Psu (P4), PDB 3RX6. The first two views in each panel are related by a 90° x-axis rotation. The first is parallel to the capsid surface, with the bottom of each structure corresponding to the parts of the protomer (denoted by arrows) involved in contacting the capsid surface. The second view is looking down towards the surface of the capsid. The last two panels show the protein fold, and a corresponding topology diagram. For each structure except the last, one protomer is colored with blue and purple indicating β-strands and α-helices within the conserved fold, while yellow and orange highlight β-strands and α-helices in non-conserved structure.

The most common structural motifs of decoration proteins are summarized in Figure 2 and Table 2. Here we consider only those structures for which high-resolution models are available in the Protein Data Bank (PDB). Many of the decoration proteins structures were novel folds when they were first determined. Novel folds appear to be more commonly represented in viruses, possibly because the proteomes of viruses are less well studied and because viral genomes are subject to higher mutation rates, affording more structural innovation [96,97].

#### 3.2.1. The β-Tulip Motif Has Three Subfamilies

The first high-resolution structure of a decoration protein was that of gpD (λ) [50]. At the time the gpD (λ) structure was a previously unobserved novel fold. The motif was named the “β-tulip” fold some 18 years later, in the context of the structurally related decoration protein gp87 (P74-26) [17]. Besides gpD (λ), gp87 (P74-26), gp88 (P23-45) and their structural homologs (Table 2), a third branch of the β-tulip fold family occurs in a domain of the head-fiber decoration protein gp8.5 (φ29) [98]. gp8.5 (φ29) has a complex elongated multi-domain structure, consisting of an N-terminal β-tulip “base” domain that contacts the capsid, and a C-terminal extended three-stranded helix-turn-helix supercoil that forms the “spike” domain emanating from the virus surface [98].

A β-tulip domain consists of a 5-stranded anti-parallel β-barrel with an α-helix intervening between strands 3 and 4. This fold is illustrated by the representative structure of gp87 (P74-26) in Figure 2A. To date most occurrences of the β-tulip folding motif have been found in virus proteins, with the exception of MoeA a molybdenum-binding protein from *E. coli* [17]. The β-tulip motif has a “bloom” side corresponding to the end of the barrel that is flared open, and a “stem” side at the opposite end that is capped by loops [17]. The β-tulip motif (blue and magenta in Figure 2A) is conjoined within a mixed α+β subdomain (yellow and orange in Figure 2A) to form a larger structure in the three subfamilies represented by gpD (λ), gp8.5 (φ29), gp87 (P74-26), and gp88 (P23-45) [17,18,51,98].

Each of the three β-tulip decoration protein subfamilies forms trimers in their capsid-bound states. The bloom side of the β-tulip interacts with the mixed α + β subdomain of the neighboring protomer to buttress the trimer [17]. Capsid-binding (illustrated by the arrows in Figure 2A) primarily involves the N-terminus of the decoration protein (‘Dec-arm’), which in the case of gp87 (P74-26) is disordered in the crystal structure but visible in the cryo-EM structure of the capsid-bound protein [28]. The N-arm of gp88 (P23-45) is also ordered when bound to the capsid [18]. This suggests the stabilization of the N-terminal segment is coupled to capsid binding. In addition to forming interactions with the capsid, the N-terminal Dec-arm also links neighboring trimers across the icosahedral three-fold and quasi-three-fold axes, forming an interlocked decoration protein chainmail surrounding the capsid [28].

#### 3.2.2. Dec (L) Has an Oligonucleotide/Oligosaccharide-Binding (OB)-Fold

The OB-fold was initially identified in proteins with oligonucleotide or oligosaccharide-binding functions but now includes proteins with functions as varied as proteinase inhibitors, chemotaxis, and molybdenum-binding proteins [99,100,101]. The fold consists of a five-stranded Greek Key β-barrel (Figure 2B) that is closed by an anti-parallel connection between strands 1 and 4 and a short parallel connection between strands 3 and 5. Typically, an α-helix between strands 3 and 4, provides a hydrophobic plug residue for one side of the β-barrel. In the Dec (L) structure [56] the OB-fold is distorted so that strands 3 and 5 are too far apart to hydrogen bond, and the α-helix is displaced relative to the axis of the barrel. The capsid-binding site in Dec (L) is comprised of the α-helix between strands β3 and β4 and two clasp-like prongs formed by the hairpin loops linking strands β1-β2 and β4-β5 (arrows in Figure 2B).

It is interesting to note that the OB-fold and β-tulip motifs are similar. Both are anti-parallel five-stranded β-barrels with an α-helix between strands β3 and β4 (Figure 2A,B). The principal difference is in the hydrogen-bonded pairing of the β-strands. It is thus conceivable that the β-tulip and OB-fold could be evolutionarily related.

As in the previously discussed examples of β-tulip proteins, the OB-fold in Dec (L) is also part of a more elaborate structure. In the Dec (L) protomers, the OB-fold is flanked by a short N-terminal strand, a short C-terminal α-helix, and a long 40 residue disordered tail [56]. The folded parts of the protomers act as the legs of a tripod that sits on the capsid [56]. Trimerization exclusively involves the 40-residue C-terminal tail, which forms a three-stranded β-helix spike in the capsid-bound structure but is unfolded in the monomers. The globular OB-fold domains are too far from each other to account for any stabilizing contacts [56]. Both the OB-fold and α-helix parts of the structure could be potential binding sites for polysaccharides [57]. This is interesting because Dec (L) has been suggested to interact with bacterial cell surfaces [13], which could be mediated through carbohydrate binding.

#### 3.2.3. Soc (T4) Has a Unique β-Tadpole Fold

**Figure 3:**
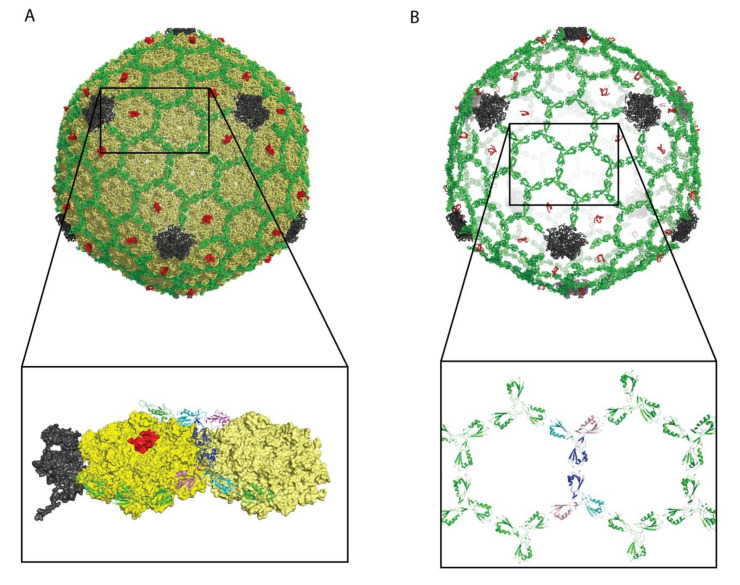
Structure of isometric T4 heads bound by Soc (T4) and Hoc (T4). The data are from PDB file 5VF3 [28]. (**A**) View of the complex: coat protein—yellow, vertex protein—grey, Soc (T4)—green, Hoc (T4)—red. Please note that only a small part of the HocT4 protein was visible in the 3.3 Å cryo-EM data. (**B**) View of the structure showing only Soc (T4) and Hoc (T4). The Soc (T4) decoration protein forms a chainmail-like structure surrounding the capsid. This is not the case for every decoration protein, for example the Dec (L) trimers in Figure 1B are isolated from each other. The expansion in (**B**) shows the Soc (T4) molecules surrounding two of the T4 hexons. For clarity two of the Soc (T4) trimers at the center of the hexons have their three protomers colored in blue, cyan, and purple.

The proteins Soc (T4) and Hoc (T4) simultaneously decorate the capsids of mature T4 and T4-like phages, with 870 Soc and 155 Hoc proteins per capsid. Both the Soc (T4) and Hoc (T4) structures were determined in Michael Rossmann’s lab [36,38,39,61]. The Soc (T4) structure was a novel fold [39]. The elongated fold called a ‘β-tadpole’ consists of a head subdomain, formed by an anti-parallel three-stranded β-sheet packed against two α-helices. A β-hairpin that extends out from strands 1 and 2 in the head subdomain, forms the tail of the tadpole. The tail subdomain is primarily involved in trimerization, while the head forms the capsid-binding site (arrows in Figure 2C). The Soc (T4) trimers act as clamps, linking neighboring capsomers in a chainmail structure that surrounds the capsid and stabilizes it against temperature and pH fluctuations [39]. To illustrate the complexity of decoration protein-capsid complexes we have chosen the Rossmann lab’s cryo-EM structure of phage T4 isometric heads complexed with Soc and Hoc, shown in Figure 3 [36].

#### 3.2.4. Hoc (T4) Has Multiple Immunoglobulin (Ig)-Like Domains

Although Soc (T4) stabilizes the capsid, Hoc (T4) has little or no effect on capsid stability but is used for cell attachment including to the T4 phage target *E. coli* [38]. Free Hoc (T4) has an elongated 4-domain structure (Figure 2D). The first three N-terminal domains have immunoglobulin (Ig)-like structures [38]. The Ig-fold consists of 7–9 anti-parallel β-strands arranged into a two-stack β-sandwich. Ig-folds are common cell-attachment modules that mediate interactions either through binding proteins or carbohydrates. A cryo-EM reconstruction showed that Hoc (T4) binds to a central depression in the T4 hexameric capsomere, as a dumbbell-shaped monomer with both Ig domain 1 and the non-Ig domain 4 contacting the capsid [61]. Unfortunately, domain 4 could not be fully seen in either the X-ray structure of the free protein nor the cryo-EM structure of capsid-bound Hoc (T4) [38,61].

#### 3.2.5. Psu (P4) Has a Unique Knotted α-Helical Fold

The α-helical decoration protein Psu (P4), which doubles as a transcription antiterminator, has a novel V-shaped knotted dimer structure (Figure 2E) [64]. Knotted protein structures are extremely rare. When they occur, they are associated with extremely high stability. This may be why this unusual motif was selected as a viral decoration protein. The C-terminal α-helix 7 (arrows in Figure 2E) is thought to be responsible for capsid-binding at the center of P4 hexameric capsomers [64].

#### 3.2.6. Additional Decoration Protein Structures

Besides the decoration proteins described above, there are examples that either are not well structurally characterized or do not fit the definition of a globular structure. These includes cases where only low-resolution structure data are currently available [66,73], proteins with substantial intrinsic disorder [25,70], and proteins that are parts of large hetero-oligomeric complexes [25,37,70].

### 3.3. Structural Homology Suggests Evolution through Horizontal Gene Transfer

Viruses and their target cells are constantly swapping genetic material through horizontal gene transfer processes leading to virus-host coevolution. Moreover, genome data suggests that typical phages are mosaics of genes generated by nonhomologous recombination of ancestral sequences [104]. Thus, horizontal transfer is likely to have occurred both between viruses and cells, and between viruses. An analysis of sequences and structures of viral capsid proteins found evidence that these probably evolved from cellular organisms on multiple occasions [105].

We performed a simple analysis to try to investigate the evolutionary origins of decoration proteins. Starting with the basis set of five well-characterized decoration protein folds (Table 2), we submitted the representative structure for each fold to a PDB-BLAST search [103] that looks for amino acid sequence homologs in the PDB database of known structures. All the hits in this search are decoration proteins with known structures that are sequence homologs of the representative set of decoration proteins (Table 2). For example, gpD (λ) was found to be a sequence homolog of SHP (P21), as previously described in the literature [52].

We next submitted the representative proteins to a DALI structural homology search [102]. This algorithm identifies structural homologs that have no sequence homology to the query structures [102]. We restricted this search to proteins that belong to phages or viruses. For example, gpD (λ) had a structural similarity hit to the PDB entry 3SUC, which is a preneck appendage protein of the phage φ29 tailspike [106]. Structural similarity between decoration and tailspike proteins has been reported previously [63,89]. In fact, the decoration protein pb10 (T5) was initially classified as a tail protein due to its predicted Ig-like domains [16]. Moreover, decoration proteins suggested to play roles in cell attachment may have evolved from tailspike proteins to facilitate host recognition [63,89]. As shown in Table 2, we find that structural homology to decoration proteins is not restricted to tailspike proteins, but occurs for a range of viral proteins including capsid and envelope proteins.

Finally, we did a DALI structural homology search restricting results to proteins that are found in the hosts of the respective phages (Table 2). For each of the decoration proteins that represent the five main folds, we found a structural homolog in the host. For example, gpD (λ) has homology to the *E. coli* molybdenum-binding protein MoeA, which as described earlier is the only example of the β-tulip fold not found in a virus. In cases where we could not find a structural homolog, probably since not all organisms are equally well-represented in the structure database, we did a BLAST search to look for sequence homologs in the host organism to one of the phage structure homologs. Thus, while gp87 (P74-26) does not have any known structural homologs in *T. thermophilus*, the structurally homologous φ29 protein 3SUC has sequence homology to a hypothetical protein in *T. thermophilus*. Taken together, the homology relationships in Table 2 suggest decoration proteins, and perhaps the cellular homologs, likely evolved through horizontal gene transfer either within phages/viruses during co-infection events, or between phages/viruses and their host organisms through recombination.

## 4. Nanotechnology Applications

**Figure 4:**
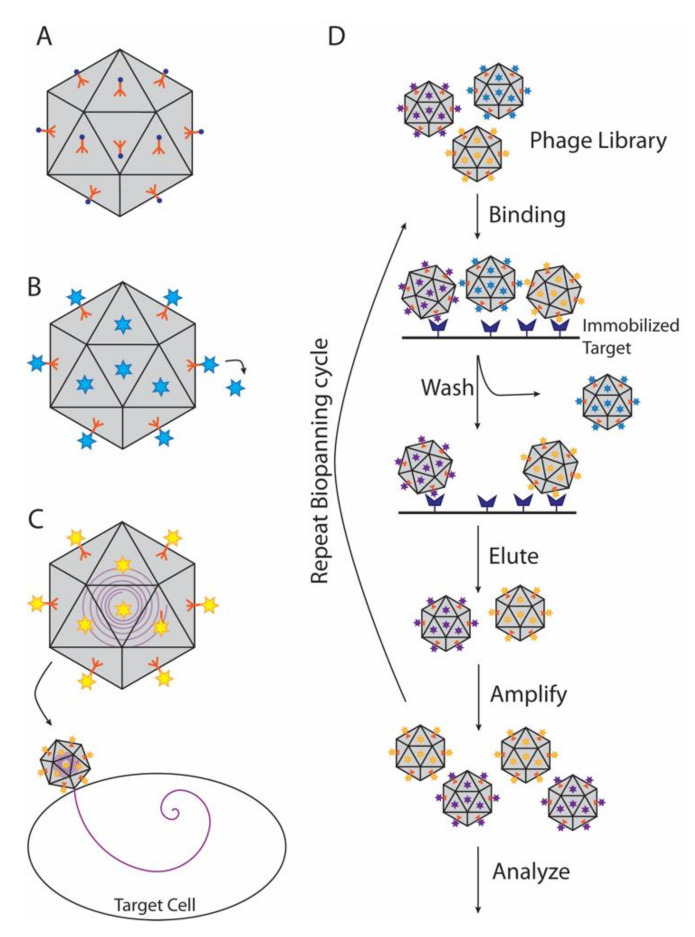
Nanotechnology applications of decoration proteins. Viral nanoparticles (VNPs) are in grey, decoration proteins in orange, and cargo molecules are shown as multi-colored circles and stars. (**A**) Functionalization of VNPs. In this example metal ions are attached to decoration proteins on VNPs, to create magnetic or conductive nanoparticles or nanowires. (**B**) A wide range of molecules can be attached to decoration proteins for phage display or cargo delivery. (**C**) Decoration proteins can be used to target VNPs carrying internal cargos to specific cells. (**D**) Biopanning can be used to find novel therapeutics.

Viruses and phages are exploited for a wide variety of uses in the fields of medicine, materials science, and nanotechnology. Among other applications, phages are being employed to treat antibiotic-resistant bacterial infections, to screen for potential drugs with phage display technology, and to deliver drugs via viral nanoparticle (VNP) vehicles [107,108]. Conjugating VNPs with moieties such as metals, polymers, or diagnostic imaging dyes is opening avenues to produce novel materials, including catalysts, biomimetics, and “smart” imaging agents (Figure 4A) [109,110,111,112,113].

### 4.1. Decoration Protein Platforms for Design of Novel Nanomaterials

Although most efforts to develop novel VNPs have focused on viral coat proteins, decoration proteins offer unique advantages. First, since decoration proteins are accessory surface molecules that do not interfere with the assembly of VNPs, they can tolerate much larger cargo molecules than coat proteins [114]. Because different decoration proteins bind at different types of symmetry sites on the icosahedral surface, it should be possible to control the patterning of cargo displayed on VNPs [92]. Stability over a range of external environments is desirable for VNPs in medical applications, especially if a drug cargo needs to be delivered orally. In this regard, the S28C mutant of the adenovirus cementing precursor protein VI, has been shown to modulate the stability of the viral capsid without impacting the infection process, thus potentially allowing for VNPs with controllable stabilities [115].

Most importantly, decoration proteins bind viruses non-covalently, making it possible to tune their binding affinities through mutagenesis or by changes in solution conditions. The non-covalent binding of decoration carrier proteins affords the opportunity to control their functionalized cargo molecule activity. For example, activity could be “turned off” by substitution of a functionalized decoration protein for the wild type, or the activity could be “swapped” by substituting a decoration protein derivatized with one type of cargo for another. Possible applications for nanomaterials with dissociable decoration protein subunits include the rational design of switchable nanomaterials such as pores that assume different diameters depending on the cargo displayed [116], multi-functionalized nanomaterials [117], nanolithography [118], and nanomaterials with temporally controlled properties [119]. Moreover, nanomaterial design need not be limited to icosahedral VNPs. Subtle changes in interactions between coat protein subunits, for example altered through site-directed mutagenesis, can divert assembly from icosahedra to other types of lattice structures such as nanotubes or nanosheets that can be similarly functionalized through decoration protein binding [92].

### 4.2. Decoration Proteins in Phage Display and Biopanning

A major application of decoration and cementing proteins is phage display (Figure 4B), a technique developed over the last four decades and highlighted by the chemistry Nobel prize in 2018 [120]. In this technique, the gene for a protein or peptide is inserted into a phage coat or decoration protein gene, producing a fusion protein that is displayed on the outside of the capsid [121,122,123]. Decoration proteins, such as Hoc (T4), Soc (T4), and IX (adenovirus), are particularly useful for display of large protein molecules or complexes in high copy numbers, which if fused to the alternative coat proteins could disrupt capsid assembly [114,123,124,125]. Thus, T4 Hoc and Soc displaying short, random peptide sequences have been used to discover additional phage proteins that bind the terminase protein, gp17, via selection method called biopanning that uses multiple rounds of screening to find molecules that bind with high affinity to chosen target (Figure 4D) [126]. Other applications include screening vaccine candidates, drug discovery, or as biosensors to detect specific antigens [127,128,129,130,131,132].

### 4.3. Decoration Proteins in Vaccine Design

Over millions of years of evolution animal immune systems have adapted to recognize viruses and virus-like particles as dangerous. Consequently, multivalent display of antigens on the surfaces of virus-like particles elicits much stronger immune responses than the corresponding free antigens [133,134]. Display of immunogenic antigens on the surfaces of VNPs through decoration protein carriers has the potential to advance rational vaccine design efforts.

Several phage display systems have been shown to stimulate an immune response in animals, including M13, λ, T7, and T4 [127]. In some systems the displayed antigen is conjugated directly to the coat protein, while in others decoration proteins were used [121,123,125]. Decoration proteins offer advantages for vaccines: (i) they can potentially accommodate larger epitopes [114]; (ii) the epitope density on the VNP surface can potentially be modulated; (iii) different molecule types can be simultaneously displayed together, such as an antigens alongside adjuvants [114,135]. Examples of decoration proteins used for vaccine development include gpD (λ), which when fused to fragments of the *Circovirus* 2 capsid protein forms the basis of a λ phage livestock vaccine for pigs [136]. In a second example, gpD (λ) fused to a prion protein from deer-stimulated production of IgA antibodies in a mouse model without the use of adjuvant [137]. VNPs displaying gpD (λ) fused to GP2, a peptide derivative from the overexpressed tumor protein HER2/nue, generated a strong cytotoxic T lymphocyte response that had anti-tumor activity when given prophylactically or therapeutically in a mouse model [138].

Hoc (T4) and Soc (T4) have the advantage of being able to carry large cargos [125,126] such as anthrax toxin oligomers with a 93 KDa molecular mass [114,139]. Additionally, Hoc (T4) and Soc (T4) were used to display fragments of the type I porin, PorA, from *Neisseria meningitides* [122], as well as the 83KDa *Bacillus anthracis* protective antigen (PA) [139,140], and the HIV protein, p24 [125] on T4 VNPs. In each case, mice immunized with these VNPs showed strong immunogenic responses to the displayed protein [122,125,139]. The use of decoration proteins to display antigens on VNPs presents new advantages for potential vaccine development.

### 4.4. Decoration Proteins used as Postmarks to Target VNP Delivery

Another potential use of surface-bound decoration proteins is to deliver cargo molecules encapsulated in VNPs to specific cell types (Figure 4C). The phage P22 system is particularly attractive as conditions for capsid assembly and cargo encapsulation are well established [6,55,92,141,142]. As mentioned earlier in the review, phage P22 can bind Dec (L) in vitro allowing cargo fused to the decoration protein to be displayed on its surface. Dec (L), which binds tightly only to expanded VNPs that mimic the mature state of phage P22, has been used to both display receptor-binding proteins and to deliver cargo molecules encapsulated in the capsid [55,92,142]. This “inside-outside” functionalization strategy demonstrates that VNPs can be simultaneously employed for both cell-targeting and payload delivery [55].

Phage T4 is likewise suitable for this application as it can hold a large volume of genetic material, and its decoration proteins are amenable for fusion with a broad range of proteins. A “progene” approach was used to simultaneously deliver both genes (encapsulated in the phage) and proteins (displayed on the surface through a Soc(T4) fusion) specifically to antigen-presenting dendritic cells via a Hoc(T4)-fused cell penetrating peptide [135]. The work shows that “inside-outside” cargo consisting of both proteins and DNA could be delivered to specific cell types through “postmarks” attached to decoration proteins. These types of approaches offer new avenues for vaccine and therapeutic strategies.

## Figures and Tables

**Table 1:** Properties and functions of decoration proteins.

Protein (Phage/Virus)	Host Organism	Structural Properties	Capsid Oligomer ^b^	Binding Symmetry ^c^	Functions	Refs
gpD (λ)	*E. coli*	β-tulip	trimer (monomer)	3F	stability, assembly	[14,48,49,50,51]
SHP (21)	*E. coli*		trimer (trimer)	3F	stability	[52]
gp56 (TW1)	*P. phenolica*		trimer	q3F	stability	[15,53]
gp87 (P74-26); gp88 (P23-45)	*T. thermophilus*		trimer	3F	stability	[17,18]
YSD1_16 (YSD1)	*S. typhimurium*		trimer	3F	stability	[54]
Tri1,2a,2b (HCMV)	*H. sapiens*		trimer	3F	stability, assembly	[21,22,23]
VP19c,23 (HSV-1)	*H. sapiens*		trimer	3F and q3F		[21,22,23]
Dec (L)	*S. enterica*	OB-fold	trimer (monomer)	3F & q3F	stability, host adhesion	[13,47,55,56,57]
Soc (T4); Soc (RB69)	*E. coli*	β-tadpole	trimer (monomer)	q2F and q3F	stability	[39,58]
Hoc (T4)	*E. coli*	Ig-like	monomer	q6F	host adhesion, phage dispersal	[36,38,59,60,61,62]
pb10 (T5)	*E. coli*		monomer	q6F	stability, host adhesion	[16,63]
gp17 (N4)	*E. coli K12*		monomer	q3F	stability, host adhesion	[19]
Psu (P4)	*E. coli*	Knotted α-helical	dimer	q6F	stability, host transcription modulation	[64,65]
gp8.5 (φ29)	*B. subtilis*	multi-domain	trimer	q3F	host adhesion	[40]
gp12 (SPP1)	*B. subtilis*	collagen-like (predicted)	trimer	q6F	host adhesion	[66,67,68]
IIIa (Adenovirus)	*H. sapiens*	4-helix bundle	complex	5F	stability, capsid ‘tape-measure’	[12,24,25,26,69]
IX (Adenovirus)		triskelion	complex	3F	stability	
VI (Adenovirus)		helical core, IDP ^a^ termini	complex	q6F	stability, endosome escape	
VIII (Adenovirus)		IDP core	complex	3F and 5F	stability	
P30 (PRD1)	Broad host specificity	extended	dimer	2F	stability, capsid ‘tape-measure’	[70,71]
P2 through P14(PCBV-1)	*C. variabilis*	variable	hexagonal lattice	variable	stability, capsid ‘tape-measure’	[37,72]
gp10 (ε15)	*S. anatum*	β-jellyroll (predicted)	dimer	2F	stability	[73]

^a^ IDP—intrinsically disordered protein; ^b^ Entries list the oligomeric state when the decoration protein is capsid-bound. In cases where the oligomerization state is known for the protein in solution, this is indicated in parentheses. For example, Soc (T4) is a trimer when capsid-bound but a monomer in solution. “Complex” denotes hetero-oligomeric interactions between multiple decoration proteins. ^c^ Abbreviations: 2F, 3F, 5F, 6F are icosahedral 2-, 3-, 5-, 6-fold symmetry centers, while imperfect quasi-symmetry centers are denoted with the letter “q”.

**Table 2:** Decoration Protein Structural Homology ^a^.

Fold	Example	PDB File	PDB-Blast Relatives ^b^	DALI Phage/Virus Homologs ^c^	Host: Host Homologs ^d^
β-tulip	gpD (λ)	*1C5E*	*1TD0*, SHP (P21)	*6QYY*, gp8.5 (φ29) *3SUC*, φ29 preneck appendage	*E. coli*: *1C5E→ 1XI8*, MoeA molybdenum biosynthesis
	gp87 (P74-26)	*6BL5*	*6I9E-H*, gp88 (P23-45)	*6XGP*, YSD1_17 major capsid protein *6QYY*, gp8.5(φ29) *3SUC*, φ29 preneck appendage *6PPB-B*, KHSV capsid vertex component	*T. thermophilus:* 3SUC → NHK40118.1, hypothetical protein
	gp8.5 (φ29)	*6QYY*	None	*2JES-A*, SPP1 portal protein *6BL5*, gp87 (P74-26), gp88 (P23-45) *1CE5*, gpD(λ)	*B. subtilis*: *2JES-A* → WP_075218525.1, hypothetical protein
OB-fold	Dec (L)	*6E3C*	None	*3QR8*, P2 membrane piercing	*S. enterica*: *6E3C→ 2OT2*, chaperone (E. coli homolog)
β-Tadpole	Soc (T4)	*3IGE*	*3IG9*, Soc (RB69)	*5VF3-A*, T4 capsid vertex protein gp24	*E. coli*: *3IG9→ 2MCF-A*, unknown function
Ig-like	Hoc (T4)	*3SHS*	*5LXK*, pb10 (T5)	*6PCI-H*, ebola spike glycoprotein *6C6Q-F*, norovirus VP1 capsid protein *6URH-H*, hepatitis C envelope glycoprotein	*E. coli:* *6PCI-H* → WP_168428099, hypothetical protein
knotted α-helix	Psu (P4)	*3RX6*	None	*1FAV-A*, HIV gp41 envelope protein	*E. coli*: *3RX6→3AJW-A*, flagellar fusion protein

^a^ PDB accession codes for protein structures are denoted in italic type; ^b^ PDB sequence homologs were identified in a PDB-Blast search of the query sequence against homologous proteins with structures available in the Protein Data Bank (PDB). Entries give the PDB accession code, followed by the name of the decoration protein. ^c^ Structural homologs in phages or viruses identified using a DALI search [102]. ^d^ Host cell homologs identified either from a BLAST sequence homology search [103] limited to proteins in the host organism, or alternatively from a DALI structural homology search [102]. The column lists the host, followed by the query decoration protein and its structural homolog when available. In cases where structure homologs are not available, a sequence homolog to a host protein is listed using its NCBI sequence accession code.
